# Improvements in cognitive performance switching from efavirenz‐ to dolutegravir‐based antiretroviral therapy in South African people with HIV: the CONNECT study

**DOI:** 10.1002/alz70855_098871

**Published:** 2025-12-23

**Authors:** Sam Nightingale

**Affiliations:** ^1^ University of Cape Town, Cape Town, South Africa

## Abstract

**Background:**

HIV can cause cognitive impairment, although it is unclear how commonly. This may partly be driven by HIV ribonucleic acid (RNA) cerebrospinal fluid (CSF) escape (ie. HIV RNA in CSF>plasma). Recently the World Health Organisation recommended first‐line HIV treatment change from efavirenz‐ to dolutegravir‐based antiretroviral therapy (ART), affecting most people with HIV globally. Efavirenz has been associated with neuropsychiatric, including cognitive, side effects; however, it is unknown whether dolutegravir offers substantial cognitive benefit.

**Method:**

178 virally supressed adults (18‐55 years) with HIV on efavirenz‐based ART completed a baseline assessment, and 145 followed‐up at 1‐year after switching to dolutegravir. 95 people without HIV were recruited from the same area, matched for age and sex, and 40 followed‐up at 1‐year. Participants completed comprehensive cognitive testing covering 7 domains. People with HIV had CSF sampling for HIV RNA quantification. Global cognition was assessed by T‐scores and low cognitive performance by global deficit score ≥0.5. Mixed effects and Poisson regression models were used to investigate the effects of switch on cognition.

**Result:**

Global cognitive performance was 2.57 T‐score points lower in people with HIV compared to those without HIV at baseline (*p* <.001), but was not significantly different between groups at follow‐up (*p* = .215). Rates of low cognitive performance were higher in people with HIV at baseline (30.1 vs. 11.7%, *p* <.001), but not different to people without HIV at follow‐up (8.28 vs. 7.50%, *p* = 1). Regression models showed people with HIV improved 1.40 points more than those without HIV (CI 0.48‐2.32, *p* = .003). There was 1 case (1.1%) of CSF HIV RNA escape at baseline and 3 (3.9%) at follow‐up; 3 of these were at low levels (CSF HIV RNA <200 copies/ml) and 1 resolved on repeat sampling without change in ART.

**Conclusion:**

Cognitive improvements likely represent efavirenz neurotoxicity. Rates of low cognitive performance in people with HIV following ART switch are lower than previously reported in an African setting. CSF HIV RNA escape was uncommon and/or transient on both treatment regimens. These data provide reassurance regarding brain health in people with HIV on modern ART, although the effect of ageing with HIV requires further exploration.

